# Interferon-λ Attenuates Rabies Virus Infection by Inducing Interferon-Stimulated Genes and Alleviating Neurological Inflammation

**DOI:** 10.3390/v12040405

**Published:** 2020-04-06

**Authors:** Yingying Li, Ling Zhao, Zhaochen Luo, Yachun Zhang, Lei Lv, Jianqing Zhao, Baokun Sui, Fei Huang, Min Cui, Zhen F. Fu, Ming Zhou

**Affiliations:** 1 State Key Laboratory of Agricultural Microbiology, Huazhong Agricultural University, Wuhan 430070, China; ying_yinglee@163.com (Y.L.); zling604@163.com (L.Z.); cuimin@mail.hzau.edu.cn (M.C.); zhenfu@uga.edu (Z.F.F.); 2 College of Veterinary Medicine, Huazhong Agricultural University, Wuhan 430070, China; zcluo2012@163.com (Z.L.); zhangyachun20063024@126.com (Y.Z.); dl505620@163.com (L.L.); z1030932572@163.com (J.Z.); baokunsui1@163.com (B.S.); dfeihuang@163.com (F.H.); 3 College of Basic Medicine, Dali University, Dali 671600, China; 4 Key Laboratory of Preventive Veterinary Medicine, College of Veterinary Medicine, Huazhong Agricultural University, Wuhan 430070, China; 5 Department of Pathology, University of Georgia, Athens, GA 30602, USA

**Keywords:** interferon-λ, rabies virus, interferon-stimulated genes, inflammation, blood-brain barrier

## Abstract

Rabies, caused by rabies virus (RABV), is a fatal neurological disease that still causes more than 59,000 human deaths each year. Type III interferon IFN-λs are cytokines with type I IFN-like antiviral activities. Although IFN-λ can restrict the infection for some viruses, especially intestinal viruses, the inhibitory effect against RABV infection remains undefined. In this study, the function of type III IFN against RABV infection was investigated. Initially, we found that IFN-λ2 and IFN-λ3 could inhibit RABV replication in cells. To characterize the role of IFN-λ in RABV infection in a mouse model, recombinant RABVs expressing murine IFN-λ2 or IFN-λ3, termed as rB2c-IFNλ2 or rB2c-IFNλ3, respectively, were constructed and rescued. It was found that expression of IFN-λ could reduce the pathogenicity of RABV and limit viral spread in the brains by different infection routes. Furthermore, expression of IFN-λ could induce the activation of the JAK-STAT pathway, resulting in the production of interferon-stimulated genes (ISGs). It was also found that rRABVs expressing IFN-λ could reduce the production of inflammatory cytokines in primary astrocytes and microgila cells, restrict the opening of the blood-brain barrier (BBB), and prevent excessive infiltration of inflammatory cells into the brain, which could be responsible for the neuronal damage caused by RABV. Consistently, IFN-λ was found to maintain the integrity of tight junction (TJ) protein ZO-1 of BBB to alleviate neuroinflammation in a transwell model. Our study underscores the role of IFN-λ in inhibiting RABV infection, which potentiates IFN-λ as a possible therapeutic agent for the treatment of RABV infection.

## 1. Introduction

Rabies causes acute incurable encephalitis and is responsible for more than 59,000 human deaths each year, thus posing a severe threat to public health [1]. Rabies virus (RABV) has a non-segmented, negative-sense RNA genome, and it is a member of the *Lyssavirus* genus in the *Rhabdoviridae* family. After a possible incubation in muscles, RABV enters neurons at the wound site, migrates to the central nervous system (CNS) via sensory and motor neurons, and spreads throughout the brain [2]. Once the symptoms appear, there is no effective treatment for rabies. Therefore, exploring potential targets for rabies treatment is very meaningful.

Innate immunity of the host is the first line of defense against viral infection [3,4]. RABV has been demonstrated to be recognized by innate immune sensors, such as retinoic acid-inducible gene I (RIG-I) or melanoma differentiation-associated protein 5 (MDA5), and induces the production of type I IFN genes (IFN-α and IFN-β) in specific cells [5,6]. Type I IFNs bind to their respective receptors, resulting in the activation of the JAK/STAT pathway [7,8]. Type III IFN is structurally and genetically similar to members of the IL-10 family of cytokines. The IFN-λ receptor IL-10Rβ is found in many cells, and the other receptor of IFN-λ, IFNLR1, is expressed preferentially on epithelial cells [9]. While type I and III IFN receptors are distantly related, they both activate the JAK/STAT1-STAT2 transcription factor pathway, which results in the formation of the interferon-sensitive responsive element (ISRE) complex that controls transcription of interferon-stimulated genes (ISGs) to inhibit viral replication [10]. ISGs, including interferon-induced protein with tetratricopeptide repeats (IFIT) 2 and Viperin, play a critical role in limiting RABV replication and spread [11,12,13].

The blood–brain barrier (BBB) is composed of tight junctions between endothelial cells of the CNS microvasculature and astrocyte end-feet. Blood–brain barrier (BBB) permeability, an important factor affecting RABV pathogenesis, has been demonstrated to be enhanced in the CNS of RABV-infected mice [14,15,16,17,18]. BBB permeability could increase in response to proinflammatory stimuli such as tumor necrosis factor-α (TNF-α), interleukin-6 (IL-6), and IFN-γ, enabling infiltration of immune effectors into the CNS to clear viral infection [19]. Although BBB permeability may be necessary to resist certain CNS infections, it is strictly regulated to avoid neurological damage caused by infiltrated inflammatory cells from the periphery [20]. IFN-λ has been reported to alleviate inflammatory responses during West Nile virus infection by limiting the permeability of the BBB [21]. One of the key mechanisms responsible for BBB breakdown is the damage of tight junctions, which are composed of transmembrane (occludin and claudins) and cytosolic (ZO-1) proteins in brain microvascular endothelial cells (BMECs) [22]. IFN-λ signaling in mouse BMECs has been demonstrated to enhance tight junction (TJ) protein localization and prevent the invasion of viruses through the BBB [21]. However, the function of IFN-λ on RABV pathogenesis is unclear. In this study, the role of IFN-λ in RABV infection was investigated, and it was found that IFN-λ could restrict RABV infection by enhancing the expression of ISGs and alleviating inflammation in the CNS by decreasing the BBB permeability.

## 2. Materials and Methods

### 2.1. Viruses, Cell Lines, Antibodies, and Animals

Recombinant RABV strain B2c was generated from CVS-B2c, which was attenuated from the challenge virus standard (CVS-24) in baby hamster kidney (BHK-21) cells [23]. The cloned cell line BSR cells were derived from BHK-21 cells [24], which were cultured in Dulbecco’s modified Eagle’s medium (DMEM) (Gibco, Grand Island, NY, USA) containing 10% fetal bovine serum (FBS) (Gibco, Grand Island, NY, USA) and 1% antibiotics (penicillin and streptomycin) (Beyotime, Wuhan, China). Mouse brain capillary endothelial cell line b.End3 cells (ATCC-CRL-2299) were cultured in DMEM containing 10% FBS and 1% antibiotics (penicillin and streptomycin). Mouse neuroblastoma (NA) cells [25] were maintained in RPMI 1640 medium (Mediatech, Herndon, VA, USA) containing 10% FBS and 1% antibiotics (penicillin and streptomycin). African green monkey kidney cells (Vero, ATCC-CCL-81) were cultured in DMEM containing 10% FBS and 2% antibiotics (penicillin and streptomycin). HEK-293T cells (ATCC-CRL-3216) were maintained in RPMI1640 medium supplemented with 10% FBS and 1% antibiotics (penicillin and streptomycin). Fluorescein isothiocyanate (FITC)-conjugated antibodies against RABV N protein were purchased from Fujirebio Diagnostics, Inc. (Malvern, PA, USA, catalog No.: 800-092) (1:500 for immunofluorescence (IF)). Rabbit anti-RABV polyclonal antibody (1:200 for western blot) and rabbit anti-RABV N monoclonal antibody (1:5000 for western blot) were prepared in our laboratory. Rabbit polyclonal anti-ZO-1 antibody was obtained from Cell Signaling Technology (Danvers, MA, USA, catalog No.: 8193S) (1:1000 for IF; 1:5000 for western blot). Rabbit polyclonal anti-β-actin antibody (Catalog No.: 8193S) (1:5000 for western blot), biotinylated goat anti-rabbit antibody (Catalog No.: 65-6140) (1:5000 for western blot), biotinylated goat anti-mouse antibody (Catalog No.: 62-6540) (1:5000 for western blot), Alexa Fluor 488-conjugated goat anti-rabbit antibody (Catalog No.: 31820) (1:500 for IF), and 4′,6-diamidino-2-phenylindole (DAPI) (Catalog No.: P36931) (1:1000 for IF) were purchased from Invitrogen (Grand Island, NY, USA). Rabbit monoclonal antibodies recognizing the phosphorylated (at Tyr701) (1:1000 for western blot) and unphosphorylated forms of STAT1 (1:2000 for western blot) were obtained from Cell Signaling Technology, Inc. (Danvers, MA, USA, catalog No.: 9167L and 14994S, respectively). Mouse polyclonal anti-CD45 antibody was obtained from Servicebio (Wuhan, P.R. China, catalog No.: GB11066) (1:3000 for IF). Rabbit anti-glial fibrillary acidic protein (GFAP) antibody was purchased from Abcam (Cambridge, MA, USA, catalog No.: ab7260) (1:200 for IF). Rabbit polyclonal anti-ionized calcium binding adaptor molecule 1 (IBA1) antibody was purchased from Proteintech (Cambridge, MA, USA, catalog No.: 10904-1-AP) (1:200 for IF). Female BALB/c mice (five or six weeks of age) and 2-day-old BALB/c mice were purchased from the Hubei Center for Disease Control, Wuhan, China. Ethical procedures for animal experiments were followed in accordance with guidelines approved by the Scientific Ethics Committee of Huazhong Agricultural University (protocol number HZAUMO-2015-029, March 29, 2015).

### 2.2. Treatment with Recombinant Murine IFN-λ

NA cells or Vero cells were seeded into 12-well plates and cultured for 24 h, and then infected with B2c at a multiplicity of infection (MOI) of 0.01. At 24 hpi, the cells were treated with 10 or 1000 ng/mL of IFN-λ2 or IFN-λ3 (BD Biosciences, San Jose, CA, USA) by addition into the culture medium. At indicated time points after treatment, virus titers in the supernatant were measured.

### 2.3. Isolation of Primary Astrocytes

Primary mixed glial cell cultures were established as described previously [26]. Briefly, brain tissues from 2-day-old BALB/c mice were dissociated by repeated pipetting and then passed through a 75-nm nylon mesh (Corning, NY, USA). The cells were washed once in cold PBS and cultured in DMEM (with high glucose) supplemented with 10% FBS and 1% penicillin–streptomycin. The medium was changed on days 3, 5, and 7. On day 10, the flasks were shaken at 260 rpm for 2 h to remove any non-adherent cells (mainly microglia). The remaining adherent astrocytes were detached with trypsin-EDTA and then plated again for further experiments. The purity of the isolated astrocytes for further studies was greater than 95%, which was examined by immunohistochemistry using the anti-GFAP antibody.

### 2.4. Isolation of Primary Microglia

Primary microglia cells were prepared from cerebral cortices of 2-day-old BALB/c mice as described previously [26]. Briefly, brain tissues were collected from the mice, and the cortex was dissected and minced in PBS containing 0.25% trypsin for digestion at 37 °C for 30 min with a shake every 5 min. After digestion, the DMEM supplemented with 5% FBS and DNase I were added and incubated for 5 min. Then the isolated cells were resuspended with DMEM supplemented with 10% FBS and the cell mass were removed by 40 μm cell sieve filtration. Finally, the isolated primary microglia cells were incubated at 37 °C for 7–9 days and change the culture medium with fresh DMEM containing 10% FBS at day 5. The purity of the isolated primary microglia for further studies was greater than 90%, which was examined by staining the cells with anti-IBA1 antibody using IFA.

### 2.5. Construction and Rescue of Recombinant RABVs Expressing Murine IFN-λ

The rRABV vector pB2c was constructed by inserting the genome of CVS-B2c into the mammalian expression vector pcDNA3.1 as described previously [27]. A transcription unit containing *BsiW*I and *Nhe*I restriction sites was inserted between the G- and L-coding sequences by deleting the pseudogene. The rB2c-IFNλ2 and rB2c-IFNλ3 cDNA clones were generated from pB2c as previously described [28]. Briefly, the pB2c vector was digested with *BsiW*I and *Nhe*I (NEB, Ipswich, MA) between the G and L genes. Murine IFNλ-2/3 cDNAs were prepared by RT-PCR amplification using template RNA isolated from VSV-infected mouse lung tissues [29]. The IFNλ-2 and IFNλ-3 genes were then inserted into pB2c, generating pB2c-IFNλ2 and pB2c-IFNλ3, respectively. PCR primers are listed in Table 1. The full length infectious clones (pB2c-IFNλ2 and pB2c-IFNλ3) and four helper plasmids (expressing genes N, P, G, and L from the parent virus B2c) were separately transfected into BSR cells using SuperFect transfection reagent (Qiagen, Valencia, CA, USA) according to procedures described in previous studies [30]. After incubating for 4 days, the culture medium was harvested and then examined for the presence of rescued rRABVs using FITC-conjugated anti-RABV N antibodies, and the specific fluorescence would be observed under an Olympus IX51 fluorescence microscope if the virus is successfully rescued.

### 2.6. Fluorescence Morphologies of rRABV-Infected Cells

Fluorescence morphologies were determined using a direct fluorescent antibody assay as previously described [30]. NA cells were infected at a low MOI (0.01) with different rRABVs, overlaid with semisolid medium containing 1% agar, and incubated at 34 °C for 48 h. The agar was removed and the adhesive cells were stained with FITC-labeled RABV N-specific antibody. Twenty fluorescent foci were examined to calculate the number of infected cells per fluorescent focus by using Image J software [31].

### 2.7. Virus Titration

Virus titers were determined using a direct fluorescent antibody assay as previously described [27]. Briefly, a series of 10-fold dilutions of the virus were prepared and used to inoculate BSR cells in 96-well microplates. The inoculations were performed in quadruplicate and then incubated at 37 °C for 48 h. After incubation, the cells were fixed with 80% ice-cold acetone and stained for 1 h with FITC-conjugated RABV N protein-specific antibodies. Antigen-positive foci were observed under an Olympus IX51 fluorescence microscope, and virus titers were calculated and presented as focus-forming units/mL (FFU/mL).

### 2.8. ELISA

ELISA was performed to quantify the amount of IFNλ-2/3 in NA cell culture supernatants. The assays were performed using commercially available mouse IFNλ-2/3 ELISA kits (RayBiotech, Atlanta, GA, USA), following the manufacturer’s instructions.

### 2.9. Quantitative Real-Time PCR (qRT-PCR)

The samples (tissues or cells) were collected on ice and homogenized in TRIzol (Invitrogen). Total RNA was isolated and used for qRT-PCR. Briefly, complementary DNA (cDNA) was prepared with 1 μg RNA as template using a first-strand cDNA Synthesis Kit (Toyobo). The thermocycler conditions were used for cDNA synthesis (10 min at 25 °C, 30 min at 55 °C, and 5 min at 85 °C). Each qPCR was conducted in duplicate with approximately 100 ng DNase-treated RNA and 5 nM primer pairs, using a one-step SYBR green qRT-PCR mix kit (Toyobo). Primers are listed in Table 2. The following conditions describe real-time PCR amplification (60 s at 94 °C; 15 s at 94 °C, 15 s at 60 °C, and 45 s at 72 °C for 40 cycles; 60 s at 72 °C), and the cycle threshold (CT) values were recorded. A standard curve was generated from serially diluted plasmids carrying a RABV N gene and the copy numbers of viral messenger RNA of RABV N gene (mRNA) and viral genome RNA (vRNA) were normalized to 1 μg of total RNA [28]. To quantify the level of vRNA, the primer vRNA-F was used for reverse transcription, while the primer N mRNA-R was used for reverse transcription of N-mRNA quantification. The CT value was inversely correlated with the mRNA concentration, and each CT unit represented a twofold change in the mRNA concentration. The mRNA levels of chemokines/cytokines and TJ proteins were normalized to β-actin mRNA levels. The results were expressed as fold change relative to mRNA levels detected in mock-infected controls.

### 2.10. Western Blot

Cell pellets were lysed in ice-cold RIPA lysis buffer containing protease inhibitor cocktail (composed of a proprietary mix of AEBSF, Aprotinin, Bestatin, E64, Leupeptin, and Pepstatin A to promote broad spectrum protection against endogenous proteases). The mixture was homogenized and centrifuged at 10,000× *g* for 10 min at 4 °C. After centrifugation, insoluble material was removed, and total protein concentration in the supernatant was measured using a BCA protein assay kit (Beyotime, Wuhan, China). Each sample was subjected to polyacrylamide gel electrophoresis, transferred onto a nitrocellulose membrane (Bio-Rad, Richmond, CA, USA), and blocked for 1 h at 37 °C with 3% bovine serum albumin (BSA) in Tris-buffered saline with 0.05% Tween-20 (TBST). Membranes were then incubated overnight with primary antibodies. After extensive washing with TBST, the membranes were incubated with secondary antibodies. Antibody binding was visualized using enhanced chemiluminescence reagents (Beyotime). Bands were quantified using ImageJ (NIH, Bethesda, MD, USA). The values represent the relative immunoreactivity of each protein, normalized to the respective loading control.

### 2.11. Pathogenicity Studies

Five-week-old female BALB/c mice (*n* = 10) were inoculated under isoflurane anesthesia. The groups received the following treatments: (a) inoculated intramuscularly (i.m.) with a 100 μL volume of 6 × 10^5^ FFU; (b) infected intradermally (i.d.) in both ears with a dose of 1.5 × 10^5^ FFU in 30 ul DMEM; (c) inoculated intranasally (i.n.) in 20 μL of a solution containing 150 FFU of B2c, rB2c-IFNλ2, or rB2c-IFNλ3 or mock-infected with DMEM. Body weight loss, clinical signs, and survivor numbers were recorded daily for 21 days. The animals were scored for clinical signs as follows: 0, normal mouse; 1, disorder movement; 2, ruffled fur; 3, trembling and shaking; 4, paralysis; 5, dead. All animals were humanely euthanized at the end of the experiment.

### 2.12. Luciferase Reporter Assay

293T cells (2×10^5^ cells per well) were seeded into 24-well plates. The cells were transfected with 50 ng of luciferase reporter plasmids (a gift of Prof. Xiao Shaobo from HuaZhong Agricultural University) [32], together with pCAGGS-IFNλ or pCAGGS, using Lipofectamine 2000 (Thermo Scientific, Shanghai, China). In parallel, 20 ng of pRL-TK *Renilla* luciferase reporter plasmid (Promega, Madison, WI, USA) was transfected to normalize transfection efficiency. Twenty-four hours after transfection, the cells were infected with B2c, rB2c-IFNλ2, and rB2c-IFNλ3 for 12 h. Luciferase activity in total cell lysates was measured using a dual-specific luciferase reporter assay system (Promega, Madison, WI, USA).

### 2.13. Quantification of Cytokine Production

Primary astrocytes were mock infected or infected with rRABVs at a MOI of 5. Cell supernatants were collected at 48 hpi. Inflammatory cytokines (IL-1β, IL-6, IL-17A, IFN-γ, KC, TNF-α, and VEGF) were quantified in the mock- and RABV-infected cell supernatants using a Quantibody Mouse Cytokine Array 1 Kit (RayBiotech, Norcross, GA, USA), according to the manufacturer’s protocol. The array was scanned using a GenePix 4000B (Molecular Devices, Axon Instruments, Silicon Valley, CA, USA). Data were collected using the GenePix Pro application at a photomultiplier tube (PMT) gain ranging from 540 to 790. A gain of 590 generated the optimal standard curve, and the results of this scan were analyzed using Q-Analyzer for QAM-CYT-1 (RayBiotech).

### 2.14. Transendothelial Permeability Assay

A transendothelial permeability assay was conducted as previously described with minor modifications [33]. B.End3 cells were cultured on Transwell filters (pore size 0.4 μm) until reaching 100% confluence. After treatments, FITC-dextran-10000 (10 kDa; Sigma-Aldrich, St. Louis, MO, USA) was applied apically at 1 mg/mL for 30 min. Samples were then removed from the lower chamber for fluorescence measurements with a fluorimeter (excitation, 492 nm; emission, 520 nm).

### 2.15. Immunohistochemistry (IHC) and Immunofluorescence

Animals were anesthetized with ether and were perfused by intracardiac injection of phosphate-buffered saline (PBS) as described previously [18]. Mouse brains were fixed with 4% paraformaldehyde for 24 h at 4 °C and then washed with PBS. Brain tissues were harvested and embedded in paraffin for coronal sections. To detect RABV, nonspecific binding was blocked with 10% goat serum, and then sections were incubated with DAPI and FITC-conjugated antibodies against the RABV N protein. To detect inflammatory cells, harvested sections were incubated with primary antibodies against CD45 at the concentrations indicated in the manufacturer’s guidelines, and then incubated with biotinylated secondary antibodies. The sections were observed under an Olympus IX51 fluorescence microscope. For immunofluorescence, b.End3 cells were seeded on coverslips. After forming a confluent monolayer, they were suspended in medium containing supernatants from infected astrocytes. The cells were subsequently fixed with 4% paraformaldehyde (PFA), permeabilized with 0.1% Triton X-100, and then incubated with rabbit anti-ZO-1 polyclonal antibodies. Finally, they were incubated with secondary antibody conjugated with Alexa Fluor 488, and with DAPI for nuclear counterstaining. Cells were imaged using a laser confocal microscope (Leica, Germany).

### 2.16. Measurement of BBB Permeability

BBB permeability was determined by measuring sodium fluorescein uptake as described previously [16,34]. Briefly, 100 μL of 100 mg/mL sodium fluorescein was injected intraperitoneally into each mouse. Peripheral blood was collected after circulation for 10 min, and PBS-perfused brains were then harvested. The recovered serum was mixed with an equal volume of 10% trichloroacetic acid (TCA) and then centrifuged for 10 min. The volume of the supernatant was adjusted to 150 μL with 5 M NaOH and 7.5% TCA. Homogenized brain samples in cold 7.5% TCA were centrifuged for 10 min at 10,000× *g* to remove debris. The supernatant was adjusted to 150 μL with addition of 5 M NaOH. Fluorescence of serum and brain homogenate samples was measured using a spectrophotometer (BioTek Instruments, VT, USA) with excitation at 485 nm and emission at 530 nm. The amount of sodium fluorescein taken up into brain tissues is calculated as (μg of fluorescence cerebrum, cerebellum, or brain stem/mg of tissue)/(μg of fluorescence sera/mL of blood) to normalize values for blood levels of the dye at the time of tissue collection. Data are expressed as fold differences between the amount of tracer in tissues from virus-infected mice and the amount in tissues from the uninfected control.

### 2.17. Statistical Analysis

All data were analyzed using GraphPad Prism 8 (GraphPad Software, lnc., San Diego, CA, USA). For the percent survival tests, Kaplan-Meier survival curves were analyzed using the log rank test. For the other data, an unpaired two-tailed *t*-test was used to determine whether differences were statistically significant. Data were representative of two independent experiments. For all results, the following notations are used to indicate significant differences between groups: *, *p* < 0.05; **, *p* < 0.01; ***, *p* < 0.001.

## 3. Results

### 3.1. IFN-λ Treatment Inhibits RABV Replication In Vitro

**Figure 1:**
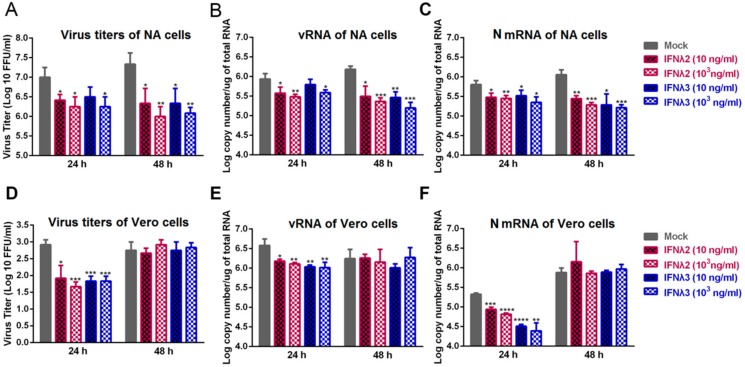
Treatment with IFN-λ inhibits RABV replication in vitro. NA or Vero cells were infected with B2c at a multiplicity of infection (MOI) of 0.01, and recombinant mouse IFN-λ2 or IFN-λ3 at 10 ng/mL and 1000 ng/mL was added to treat the B2c infected NA or Vero cells at 24 h post infection (hpi). Cell supernatants were harvested at 24 h and 48 h after the treatment for virus titration on NA cells (**A**) or Vero cells (**D**). The production of vRNA in NA cells (**B**) or Vero cells (**E)** was measured by qRT-PCR. RABV N transcription levels in NA cells (**C**) or Vero cells (**F)** were determined by qRT-PCR. Error bars represent the standard error (SE, *n* = 3). The following notations were used to indicate significant differences between groups: *, *p* < 0.05; **, *p* < 0.01; ***, *p* < 0.001; ****, *p* < 0.0001.

To test whether IFN-λ restricts RABV replication in vitro, NA or Vero cells were infected with B2c strain, and recombinant mouse IFN-λ2 or IFN-λ3 at 10 ng/mL and 1000 ng/mL were added to treat the RABV infected cells at 24 h post infection (hpi), and the virus titers, the levels of mRNA of RABV N gene (N mRNA) and viral RNA (vRNA) were measured at 24 and 48 h after the treatment. In NA cells, as shown in Figure 1A–C, the addition of IFN-λ2 or IFN-λ3 reduced the virus titers and the levels of N mRNA and vRNA at both time points. Notably, at 48 h after treatment with 1000 ng/mL of IFN-λ2 or IFN-λ3, more than 10-fold decreases of virus titers were observed compared with mock-treated cells. In the Vero cells, an IFN-α/β independent cell line, the addition of IFN-λ2 or IFN-λ3 reduced the virus titers and the levels of N mRNA and vRNA at 24 h post treatment as shown in Figure 1D–F. At 48 h post treatment, no significant difference was observed in the virus titers and the levels of N mRNA and vRNA among all. These results suggest that IFN-λ2 and IFN-λ3 inhibits RABV replication in the two cell lines used.

**Figure 2:**
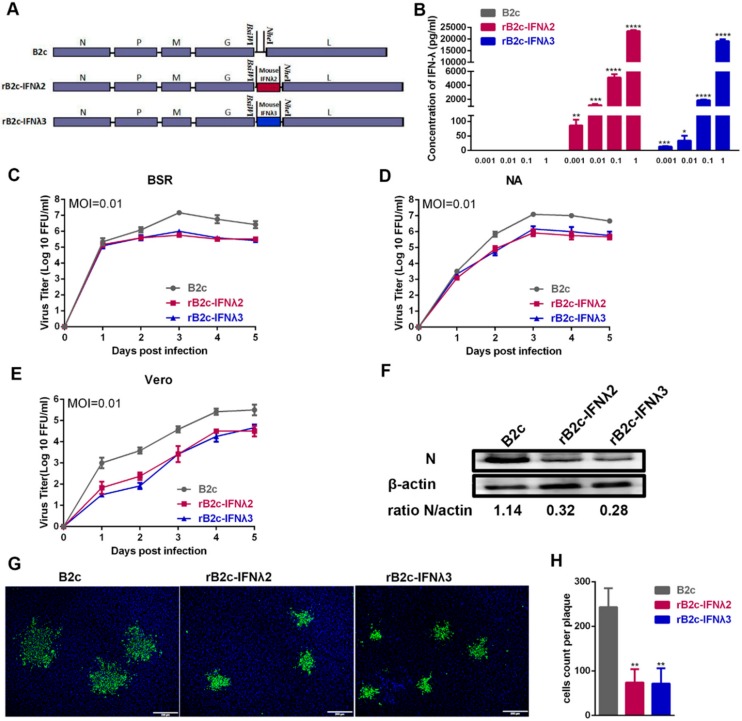
Construction and characterization of recombinant RABVs expressing IFN-λ2 or IFN-λ3. (**A**) Schematic diagram describing the construction of B2c, rB2c-IFNλ2, and rB2c-IFNλ3. The pcDNA3.1-B2c plasmid was derived from CVS-B2c by deleting the long non-coding region of the G gene and adding *BsiW*I and *Nhe*I sites between the G and L genes. Murine IFN-λ2 or IFN-λ3 coding sequences were then inserted into the RABV genome between the G and L genes. (**B**) Expression of IFN-λ2 and IFN-λ3 was measured by by ELISA. Briefly, NA cells were infected with B2c, rB2c-IFNλ2, or rB2c-IFNλ3 (MOI = 1, 0.1, 0.01, or 0.001) for 24 h, and the cell culture supernatants were then harvested to determine the quantity of murine IFN-λ2 or IFN-λ3 using a commercial ELISA kit. (**C**) BSR, NA (**D**), or Vero cells (**E**) were infected with different rRABVs at a MOI of 0.01, and multiple-step growth curves were depicted according to the viral titers at different time points. Error bars represent the SE (*n* = 3). (**F**) RABV N protein expression in NA cells after infection with rRABVs. N protein was detected by western blot at 48 hpi and ratios of N/β-actin were calculated using ImageJ. (**G**) Comparison of the fluorescence morphologies of NA cells infected by different rRABVs at 48 hpi. Twenty fluorescent foci were examined to determine the number of infected cells per fluorescent focus (**H**). Scale bars represent 200 μm. Error bars represented the SE (*n* = 3). The following notations were used to indicate significant differences between groups: *, *p* < 0.05; **, *p* < 0.01; ***, *p* < 0.001; ****, *p* < 0.0001.

To further characterize the role of IFN-λ in RABV infection in the mouse model, recombinant RABVs (rRABVs) expressing murine IFN-λ2 or IFN-λ3, designated as rB2c-IFNλ2 and rB2c-IFNλ3 respectively, were constructed as shown in Figure 2A, and rescued as described previously [27]. The insertion of both genes into the RABV genome was verified by RT-PCR and sequencing (data not shown). To detect whether the rRABVs could express IFN-λ2 or IFN-λ3, NA cells were infected with the rRABVs, and IFN-λ2 or IFN-λ3 were determined by ELISA. As shown in Figure 2B, ELISA results indicate that IFN-λ2 and IFN-λ3 were well expressed in rB2c-IFNλ2 and rB2c-IFNλ3 infected cells, respectively, in a dose-dependent manner, whereas those expressed in B2c did not. Moreover, viral growth curves on BSR, NA, and Vero cells were depicted. As shown in Figure 2C–E, compared with parent virus B2c, more than 10-fold losses of viral titers were observed in rB2c-IFNλ2 or rB2c-IFNλ3 infected BSR or NA cells at 72 and 96 hpi, and in rB2c-IFNλ2 or rB2c-IFNλ3 infected Vero cells at all tested time points. Additionally, consistent with the results of the growth curve, western blot experiments detecting RABV N protein showed that expression of IFN-λ2 or IFN-λ3 by the rRABVs reduced RABV N protein levels in infected NA cells at 48 hpi (Figure 2F). Furthermore, the effects of expression of IFN-λ2 or IFN-λ3 on virus spread were measured by observing the fluorescence morphologies of rRABV-infected cells and counting the number of RABV-positive cells using fluorescence microscopy. As a result, the fluorescence foci of cells infected with rB2c-IFNλ2 or rB2c-IFNλ3 were significantly smaller than those infected with B2c (Figure 2G,H), indicating that expression of IFN-λ suppresses the cell-to-cell spread of RABV. Taken together, these results suggest that expression of IFN-λ2 or IFN-λ3 inhibits RABV replication and spread in infected cells.

### 3.2. IFN-λ Attenuates the Pathogenicity of RABV In Vivo

**Figure 3:**
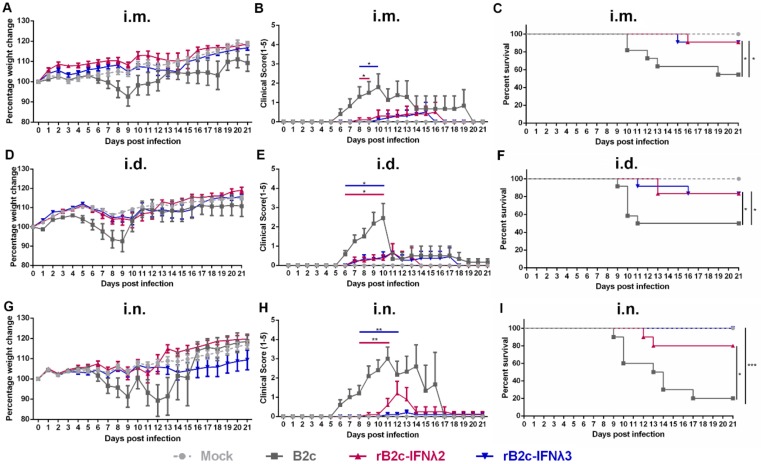
IFN-λ attenuates rRABV pathogenicity in mice. Five-week-old female BALB/c mice were inoculated i.m. (6 × 10^5^ FFU), i.d. (1.5 × 10^5^ FFU), or i.n. (150 FFU) with B2c, rB2c-IFNλ2, rB2c-IFNλ3, or DMEM alone. Body weight loss (**A**,**D**,**G**), clinical signs of rabies assessed by scores (**B**,**E**,**H**), and mortality rates (**C**,**F**,**I**) were observed daily for 21 days. Error bars represent the SE (*n* = 10). The following notations were used to indicate significant differences between groups: *, *p* < 0.05; **, *p* < 0.01; ***, *p* < 0.001.

To further investigate whether IFN-λ restricts RABV infection in vivo, groups of five-week-old female BALB/c mice were mock-infected with DMEM or inoculated with B2c, rB2c-IFNλ2, or rB2c-IFNλ3 by different routes. The body weight losses of mice infected with rB2c-IFNλ2 or rB2c-IFNλ3 by the three routes (i.m., i.d., or i.n.) were lower than those infected with B2c, as shown in Figure 3A,D,G. Similarly, the clinical scores of mice infected with rB2c-IFNλ2 or rB2c-IFNλ3 by either route were lower than those infected with B2c (Figure 3B,E,H). Consistently, significantly higher percentage of survivor ratios were observed in rB2c-IFNλ2 or rB2c-IFNλ3 infected mice than B2c infected mice (Figure 3C,F,I). It is worth noting that the most striking enhancement on survival rates among the three infection routes were observed in i.n. route—that 100% and 80% of mice i.n. infected with rB2c-IFNλ3 (*p* = 0.0005) and rB2c-IFNλ2 (*p* = 0.0126), respectively, survived, while 80% of B2c i.n. infected mice died of rabies within 17 days (Figure 3I). All the mice exhibited RABV-related symptoms and death were confirmed by RT-PCR from brain tissues.

**Figure 4:**
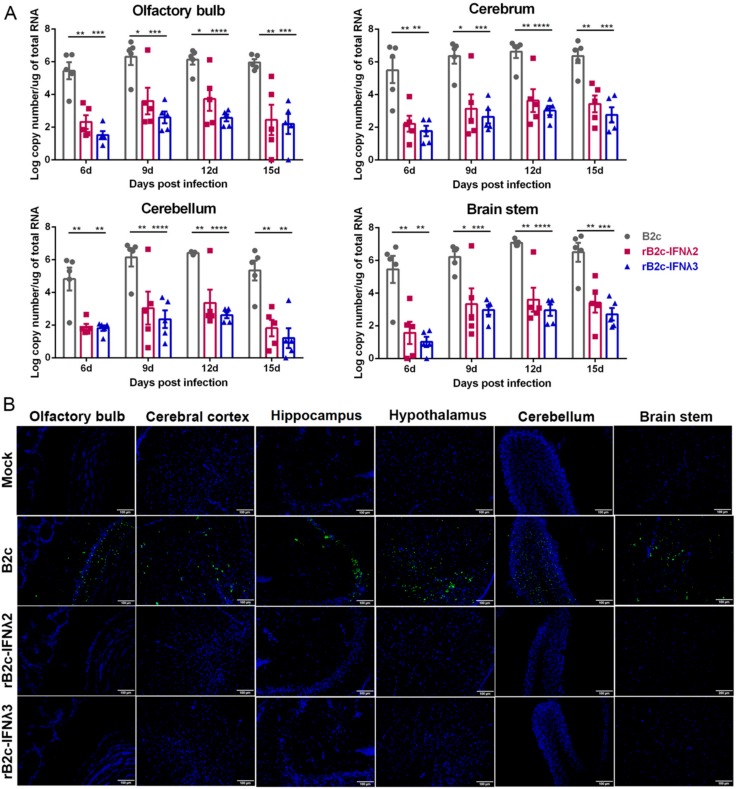
IFN-λ restricts RABV replication in the mouse brain. Five-week-old female BALB/c mice were inoculated i.n. with 150 FFU of B2c, rB2c-IFNλ2, rB2c-IFNλ3, or DMEM alone. (**A**) N mRNA copy number was measured in mouse olfactory bulb, cerebrum, cerebellum, and brain stem at 6, 9, 12, and 15 dpi by qRT-PCR (*n* = 5). (**B**) Mouse brain sections (*n* = 3) were stained with FITC-labeled RABV N-specific antibody to detect the distribution of viral antigen in the central nervous system (CNS) parenchyma (shown in green); nuclei stained with 4’,6-diamidino-2-phenylindole (DAPI) are shown in blue. Representative images were acquired at 40× magnification (scale bar = 100 μm). Error bars represent the SE. The following notations were used to indicate significant differences between groups: *, *p* < 0.05; **, *p* < 0.01; ***, *p* < 0.001; ****, *p* < 0.0001.

To investigate the viral load in the brains of infected mice, BALB/c mice were i.n. infected with 150 FFU of rRABVs, or mock-infected with DMEM, and viral burdens were analyzed in different parts of the brain by qRT-PCR at 6, 9, 12, and 15 days post infection (dpi). Viral copy numbers were extrapolated by quantitating mRNAs corresponding to the RABV N gene. As expected, viral copy numbers in the olfactory bulb, cerebrum, cerebellum, and brain stem of rB2c-IFNλ2 or rB2c-IFNλ3 infected mice were significantly lower than those infected with B2c (Figure 4A). Additionally, viral antigen (RABV N protein) in different brain tissues was also detected using immunofluorescence staining. Consistent with the qRT-PCR results, almost no (or under detection limit) viral antigens were observed in all the parts of the brains of rB2c-IFNλ2 or rB2c-IFNλ3 infected mice, while an obviously positive fluorescent signal was observed in all parts of the brains of detected mice that were infected with B2c (Figure 4B). All the above data demonstrate that IFNλ significantly reduces the pathogenicity of RABV in mice.

### 3.3. IFN-λ Limits RABV Replication by Enhancing the Expression of ISGs

**Figure 5:**
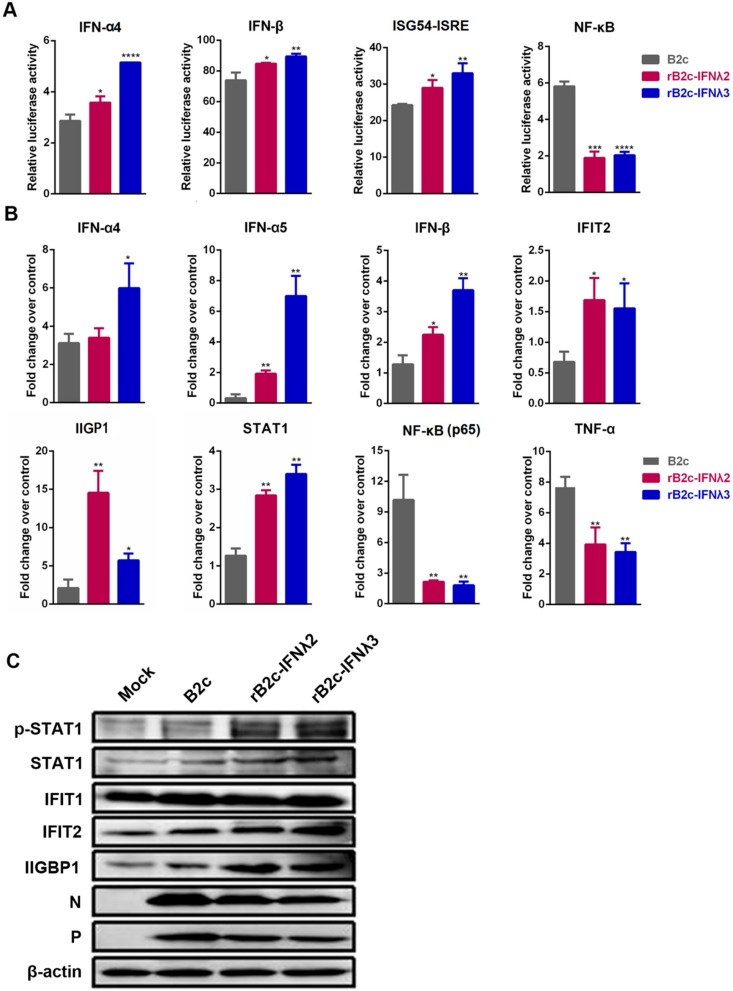
IFN-λ activates STAT1/2 and enhances the production of ISGs. (**A**) NA cells were transfected with plasmids encoding IFN-α4, IFN-β, ISG54-ISRE or NF-κB firefly luciferase reporter, respectively. After 24 h, cells were left uninfected or were infected for 12 h with different rRABVs. Luciferase assays were performed to analyze the promoter activity of ISRE, Ifn-α4, Ifn-β and NF-κB, respectively. Luciferase reporter activity was expressed as fold change that normalized to Renilla luciferase activity. (**B**) NA cells were infected with B2c, rB2c-IFNλ2, or rB2c-IFNλ3 at a MOI of 1. At 24 hpi, total RNA was isolated and Ifn-α4, Ifn-α5, Ifn-β, STAT1, IFIT2, IIGP1, NF-κB (p65), and TNF-α mRNA levels were analyzed by qRT-PCR. (**C**) Protein levels of STAT1, IFIT1, IFIT2, IIGP1, and β-actin in different rRABV-infected NA cells were measured by western blot at 24 hpi. Error bars represent the SE (*n* = 3). The following notations were used to indicate significant differences between groups: *, *p* < 0.05; **, *p* < 0.01; ***, *p* < 0.001; ****, *p* < 0.0001.

As a typical neurotropic virus, RABV mainly infects neurons in the brain. Therefore, to define the signaling pathway by which IFN-λ restricts RABV infection, a luciferase reporter assay was carried out in NA cells as described in the Methods section. The results suggest that expression of IFN-λ2 or IFN-λ3 could activate IFNα4, IFNβ, and ISG54-ISRE, but down-regulated the expression of nuclear factor κB (NF-κB) (Figure 5A). Furthermore, the mRNA levels of IFN-α, IFN-β, STAT1, interferon-induced protein with tetratricopeptide repeats 2 (IFIT2), and IFN-inducible GTPase 1 (IIGP1) in different rRABV infected NA cells were detected by qRT-PCR. Significantly higher levels of IFN-α4, IFN-α5, IFN-β, STAT1, IFIT2, and IIGP1 were detected in the cells infected with rB2c-IFNλ2 or rB2c-IFNλ3 than those in cells infected with B2c. Meanwhile, the mRNA levels of NF-κB (p65) and TNF-α were significantly decreased in NA cells infected with rB2c-IFNλ2 or rB2c-IFNλ3 compared with those infected with B2c (Figure 5B). Additionally, expression of ISGs (IFIT2 and IIGP1) and activation of JAK-STAT pathway, phosphorylation of STAT1, were also measured by western blotting assay at 24 hpi, and high levels of phosphorylated STAT1, IFIT2 and IIGP1 were detected in rB2c-IFNλ2 or rB2c-IFNλ3 infected cell. These data demonstrate that IFN-λ enhances the expression of ISGs via activating JAK-STAT pathway, and thus inhibits RABV replication.

### 3.4. IFN-λ Avoids the Production of Excessive Inflammatory Cytokines

**Figure 6:**
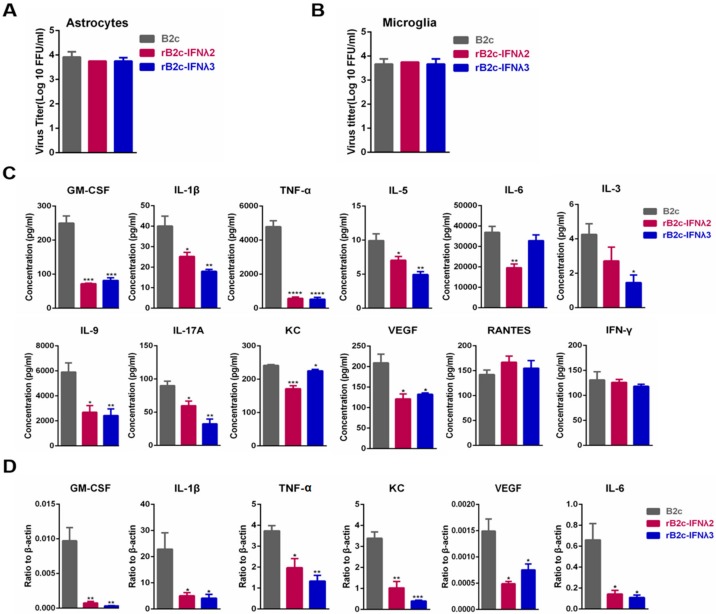
IFN-λ reduces the production of inflammatory cytokines in primary astrocytes and microglia cells. Astrocytes and microglia cells isolated from 2-day-old suckling mice were infected with B2c, rB2c-IFNλ2, or rB2c-IFNλ3 at a MOI of 5, and cell supernatants and lysates were collected at 48 hpi for astrocytes and microglia cells, respectively, for the determination of indicated cytokines. The rRABV titers were measured using a focus-forming assay and are expressed as FFU/mL on astrocytes (**A**) and microglia cells (**B**). Concentrations of the indicated cytokines in astrocyte supernatants were measured using a cytokine array (**C**) and microglia cells lysates were measured by qRT-PCR (**D**). Error bars represent the SE (*n* = 3). The following notations were used to indicate significant differences between groups: *, *p* < 0.05; **, *p* < 0.01; ***, *p* < 0.001; ****, *p* < 0.0001.

As is known, astrocytes and microglia cells play an important role in innate immunity and neuroinflammation in the CNS. Hence, primary astrocytes and microglia cells were isolated as described in the Materials and Methods section and infected with different rRABVs at a MOI of 5, and the production of several inflammatory cytokines was measured in the infected astrocytes and microglia cells. Viral titers in the supernatants of astrocytes or microglia cells incubated with B2c, rB2c-IFNλ2, or rB2c-IFNλ3 were nearly equal (Figure 6A,B). The supernatants of astrocytes were then applied to a protein array to quantify the production of specific cytokines, and the mRNA levels of GM-CSF, IL-1β, IL-6, KC, TNF-α, and VEGF were quantified in microglia cells by qRT-PCR. As shown in Figure 6C, pro-inflammatory cytokines, including TNF-α, IL-6, IL-17A, IL-1β, chemokine (C-X-C motif) ligand 1 (CXCL1/KC), and vascular endothelial growth factor (VEGF, which is related to BBB opening), were significantly lower in astrocytes infected with rB2c-IFNλ2 or rB2c-IFNλ3 than those infected with B2c. Similarly, the mRNA levels of GM-CSF, IL-1β, IL-6, KC, TNF-α, and VEGF were significantly decreased in microglia cells infected with rB2c-IFNλ2 or rB2c-IFNλ3 than those infected with B2c (Figure 6D). Together, these results suggest that IFN-λ represses the production of inflammatory cytokines induced by RABV infection.

### 3.5. IFN-λ Restricts Infiltration of Inflammatory Cells into the CNS by Decreasing BBB Permeability

**Figure 7:**
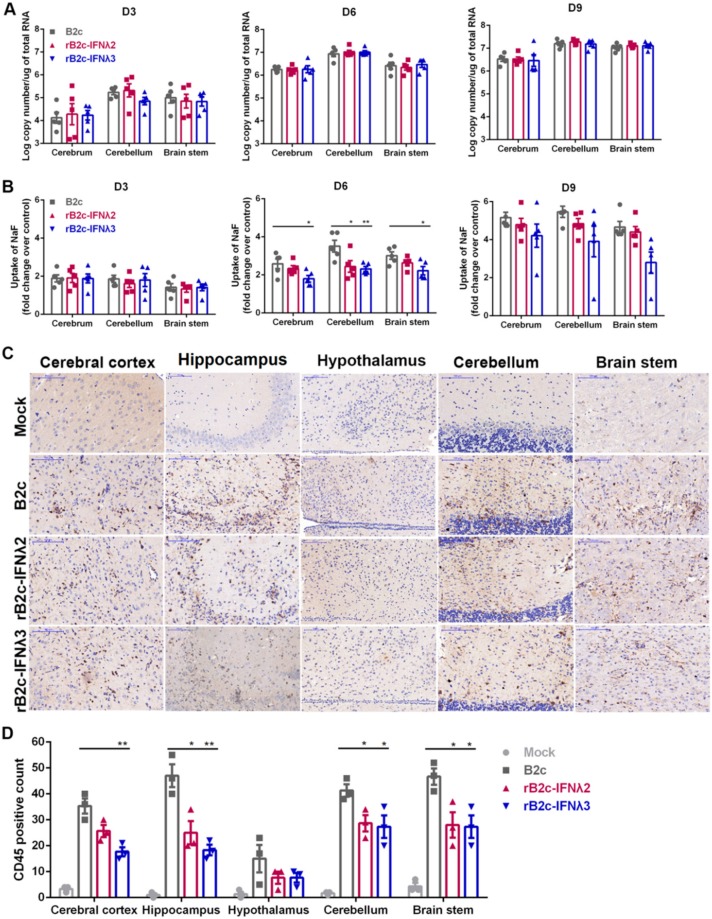
IFN-λ decreases BBB permeability and alleviates neurologic inflammation in the mouse brain. Six-week-old female BALB/c mice were inoculated i.c. with 100 FFU of rB2c, rB2c-IFNλ2, rB2c-IFNλ3, or DMEM (mock). (**A**) At 3, 6, and 9 dpi, mice (*n* = 5) were euthanized, and the brains were harvested, and viral loads were determined by qRT-PCR. (**B**) The change in BBB permeability was assessed by measuring the amount of sodium fluorescein uptake in the cerebrum, cerebellum and brain stem using a spectrophotometer after intraperitoneal administration at 3, 6, and 9 dpi (*n* = 5). (**C**) For the assessment of neurologic inflammation, six-week-old female BALB/c mice were inoculated i.c. with 100 FFU of B2c, rB2c-IFNλ2, rB2c-IFNλ3, or DMEM (mock). At 6 dpi, mice brains were collected and embedded in paraffin. The sections were then stained with anti-CD45 antibody to quantify inflammation. The scale bars represent 100 µm (*n* = 3). (**D**) CD45^+^ lymphocytes from at least three different randomly selected areas of each brain region were counted. Error bars represent the SE. The following notations were used to indicate significant differences between groups: *, *p* < 0.05; **, *p* < 0.01.

It is possible that the expression of IFN-λ in the CNS can subdue the neuroinflammatory response and block the elevation of BBB permeability due to the low expression level of inflammatory cytokines and VEGF in rB2c-IFNλ2 or rB2c-IFNλ3 infected astrocytes. Meanwhile, the enhancement of BBB permeability that associated with RABV infection is caused by cytokines and the infiltration of inflammatory cells, as demonstrated in a previous study [16]. To test this hypothesis, six-week-old female BALB/c mice were i.c. infected with rRABVs. To exclude the effect of different virus loads on the regulation of BBB permeability and neuroinflammation, the brains of mice infected by different rRABVs were harvested to analyze viral burden by qRT-PCR. At 3, 6, and 9 dpi, mRNA levels of RABV N were nearly equal in different brain regions of mice infected by B2c, rB2c-IFNλ2, or rB2c-IFNλ3 (Figure 7A). Sodium fluorescein was injected into the mice via the tail vein for measurement of BBB permeability. At 3 and 9 dpi, no significant difference in the amount of sodium fluorescein uptake was detected among the three groups. At 6 dpi, leakage of sodium fluorescein from the peripheral circulation into the cerebrum, cerebellum, and brain stem in B2c-infected mice was significantly higher than those in mice infected with rB2c-IFNλ2 or rB2c-IFNλ3 (Figure 7B). Moreover, the brain sections were then stained with anti-CD45 antibody to observe and quantify the infiltration of CD45^+^ cells induced by different rRABVs infection. Less positive signal was observed in different parts of brain from mice infected with rB2c-IFNλ2 or rB2c-IFNλ3 than those from mice infected with B2c (Figure 7C). Significantly more CD45^+^ lymphocytes were found in the cerebral cortex, hippocampus, hypothalamus, cerebellum, and brain stem from the mice infected with B2c than those infected with rB2c-IFNλ2 or rB2c-IFNλ3 at 6 dpi (Figure 7D). These results indicate that IFN-λ declines the BBB permeability to prevent excessive infiltration of inflammatory cells into the CNS during RABV infection.

### 3.6. IFN-λ Maintains Endothelial Barrier Integrity

**Figure 8:**
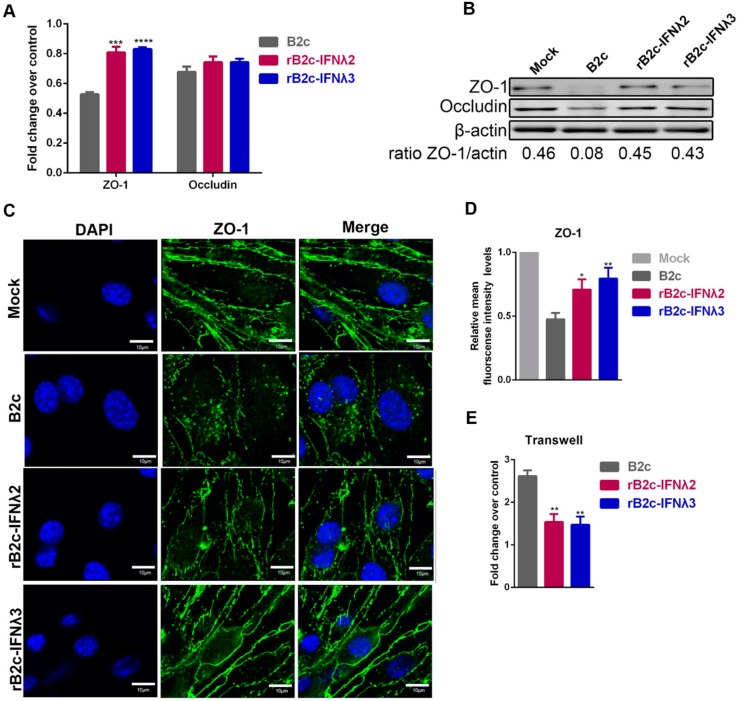
IFN-λ maintains protein expression in tight junctions in b.End3 cells. Mouse brain capillary endothelial (b.End3) cells were co-cultured for 24 h with extracts from supernatants of astrocytes that had been mock-infected with DMEM or infected with B2c, rB2c-IFNλ2, or rB2c-IFNλ3 at a MOI of 5. Expression of ZO-1 and Occludin was detected by qRT-PCR (**A**) and western blot (**B**). The integrity of TJ protein ZO-1 was measured by confocal microscopy (**C**). Relative mean fluorescence intensity for the regions of interest (ROI) was determined using Image J (**D**). B.End3 cells were cultured on transwell inserts and treated with extracts from supernatants of astrocytes that had been mock infected with DMEM or infected with B2c, rB2c-IFNλ2, or rB2c-IFNλ3 at a MOI of 5. At 24 hpi, FITC-dextran-10000 was added into the upper insert of the transwell and then the permeation of FITC-dextran-10000 into the lower chamber was measured using a fluorimeter (excitation, 492 nm; emission, 520 nm) (**E**). Error bars represent the SE (*n* = 3). The following notations were used to indicate significant differences between groups: *, *p* < 0.05; **, *p* < 0.01; ***, *p* < 0.001; ****, *p* < 0.0001.

To explore the mechanism by which IFN-λ reduces BBB permeability, a transwell model was performed. Mouse brain capillary endothelial (b.End3) cells were cultured in the upper chamber of a transwell insert, with supporting astrocytes in the lower chamber. The b.End3 cells were then treated for 24 h with extracts from the supernatants of astrocytes infected with different rRABVs, and the expression of the TJ proteins ZO-1 and occludin were then analyzed by qRT-PCR and western blot. Both mRNA (Figure 8A) and protein expression (Figure 8B) of ZO-1 in cells treated with supernatants from B2c-infected astrocytes was significantly lower than that those incubated with the supernatants from rB2c-IFNλ2 or rB2c-IFNλ3 infected astrocytes. Additionally, supernatant-treated cells were also stained with anti-ZO-1 polyclonal antibodies and imaged by a laser confocal microscope to assess the integrity of ZO-1. The rB2c-infected astrocytes supernatants caused dissociation of ZO-1, while those treated with rB2c-IFNλ2 or rB2c-IFNλ3 infected astrocytes supernatants reduced the effect (Figure 8C,D). To further evaluate the integrity of the endothelial monolayer, FITC-dextran 10,000 was added to the upper chamber of a transwell insert and the fluorescence in the lower chamber was monitored. As shown in Figure 8E, the leakage of FITC-dextran 10,000 was significantly lower when treated with supernatants from rB2c-IFNλ2 or rB2c-IFNλ3 infected astrocytes than that treated with B2c infected astrocytes supernatant. These data suggest that IFN-λ maintains the integrity of TJ proteins, resulting in the decrease in the BBB permeability during RABV infection.

## 4. Discussion

IFN-λ signaling through the IFNLR1 receptor on intestinal epithelial cells (IECs) induces antiviral effectors such as ISGs via STAT1/STAT2/IRF9-mediated transcription, thereby boosting defenses against intestinal viruses such as rotavirus, reovirus, and norovirus [35,36,37,38]. The neurotropic West Nile virus (WNV) is inhibited in the CNS by IFN-λ through a mechanism that modulates endothelial cell tight junction integrity [21]. In our study, we found that IFN-λ curtails the replication of another neurotropic virus, RABV, in cells. Moreover, IFN-λ also reduces RABV pathogenicity in infected mice by different infection routes, especially i.n. route. IFN-λ has been reported to be particularly important for innate pathogen defense at mucosal barriers [39], which provides a possible explanation for a better survivor rate in rRABV-expressing IFN-λ-infected mice by i.n. route. In addition, IFN-λ is expressed in a tissue-specific manner and its receptor IFNLR1 exhibits a much more tissue-specific expression pattern, with a preference for epithelial cells [10,40]. During intranasal challenge with RABV, the expression of IFNLR1 by nasal epithelial cells is necessary for the responsiveness of the tissue to IFN-λ and the suppression of viral replication in nasal mucosa. Therefore, IFN-λ has evolved as a specific factor that can prevent the invasion of some neurotropic viruses through nasal epithelial cells. The ability of IFN-λ to induce ISG expression in a targeted set of epithelial cells also suggests that IFN-λ promotes a focused antiviral or immunomodulatory response [41,42].

The main antiviral functions of IFN-λ have been linked to the activation of the STAT1/2 signaling pathway [43,44]. Activation of STAT1/2 is required for optimal transcription of ISGs, which establish antiviral defenses. IFN-α/β is also engaged in antiviral processes. Cooperation between IFN-α/β and IFN-λ further increases STAT1/2-activation. In our study, ISRE activity was upregulated by the expression of IFN-λ during RABV infection, indicating that IFN-λ plays a positive role in activating ISRE, which is involved in the downstream production of ISGs [45]. ISGs such as IFIT2 and IIGP1 were reported to restrict RABV replication [13,46]. The two ISGs were elevated after the infection of rRABVs expressing IFN-λ in our study, indicating that IFN-λ could activate the STAT1/2 signaling pathway during RABV infection, resulting in the downstream production of ISGs to restrict RABV infection.

Encephalitis induced by lab-attenuated RABV is characterized by obvious CNS inflammation [18,47,48]. It has previously been reported that RABV induced inflammation in microglia cells mainly through p38 and NF-κB pathways [49]. The results of luciferase reporter assay in our study implied that IFN-λ expression could suitably suppress the activation of NF-κB. Subsequently, production of pro-inflammation cytokines was remarkably decreased, as expected. Consistent with these observations, one previous study showed that excessive expression of IFN-λ caused by IAV infection, whereas the degradation of IκB and the activation of NF-κB was significantly decreased in the infected cells. In contrast, disruption of IFN-λ signaling pathway resulted in the activation of NF-κB [50]. On the other hand, IFN-λ that is induced in DCs and macrophages does not augment proinflammatory cytokine production during viral infection [51]. Type I IFN provides antiviral resistance but also induces proinflammatory responses essential for countering infection [52]. Previous study demonstrated that IFN-λ could upregulate suppressor of cytokine signaling 1 (SOCS1) and SOCS3, which reduce the devastating effects of excessive inflammation [53]. Consistently, in our study, it was found that IFN-λ could decrease the NF-κB activation and further alleviate inflammation via suppression of neutrophil infiltration and proinflammatory cytokines such as TNF-α, IL-17A, IL-1α, IL-1β, IL-6, IL-12, and VEGF during RABV infection, all of which contribute to BBB breakdown. The role of IFN-λ in NF-κB activation and inflammation production in the context of RABV infection appears to be important, but the detailed mechanism of this process remains to be fully explored.

Although IFN-λ decreases expression of proinflammatory cytokines, it does not require the activation of inflammation to modulate BBB permeability [51]. A previous study demonstrated that STAT1/2 signaling or protein synthesis is not required for endothelial barrier tightening [21]. Indeed, in our study, IFN-λ signaling was found to maintain the expression of TJ protein ZO-1 and protect its integrity, which tightens the endothelial barrier. The tightened barrier also prevents RABV transit across epithelial surfaces, such as the nasal mucosa barrier, which could be another explanation for the strongest attenuation of RABV pathogenicity after i.n. infection.

It was reported that approximately 6 kb of DNA at gene loci for IFNL2 and IFNL3 have nucleotide sequence identity greater than 97% for the whole genomic region in both human and mouse, indicating that the function of IFN-λ2 and IFN-λ3 could be very similar [54]. Consistently, the results in affecting RABV replication and pathogenicity are similar between rB2c-IFNλ2 and rB2c-IFNλ3 in our study. Additionally, non-murine cell line Vero cells, which are supposed to be an IFN-α/β independent cell line, were also used in our study to exclude the possible involvement of type I IFN during the IFN-λ inhibiting RABV replication. It has been reported that both mouse IFN-λ2 and IFN-λ3 were capable of up-regulating MHC class I antigen expression in several human cell lines and induced antiviral protection in mouse B16 cells or human HT29 cells infected with VSV [55]. These data indicate that the mouse IFN-λ could potentially be functional in cells derived from species other than mouse. In conclusion, our data indicate that IFN-λ, either IFN-λ2 or IFN-λ3, can restrict RABV infection by inducing ISGs, limiting blood–brain barrier permeability to decrease the neuroinflammation, and thus attenuating the pathogenicity of RABV.

## Figures and Tables

**Table 1:** Primers for construction and rescue of recombinant rabies viruses (RABVs) expressing murine IFN-λ.

Primers	Sequence (5′–3′)
IFNλ-*BsiW*I-F	TTGCGTACGATGCTCCTCCTGCTGTTG
IFNλ-*Nhe*I-R	CTAGCTAGCTCAGACACACTGGTCTCC
B2c-GL-F	CAGGGGGGAATGTGTCAGTC
B2c-GL-R	GCCTCTGACTCAATTGGATC

*BsiWI* and *NheI* sites are underlined.

**Table 2:** Primers for qRT-PCR.

Primers	Sequence (5′–3′)
N mRNA-F	GATCGTGGAACACCATACCC
N mRNA-R	TTCATAAGCGGTGACGACTG
vRNA-F	CTCCACAACGAGATGCTCAA
vRNA-R	CATCCAACGGGAACAAGACT
IFN-a4-F	CCCACAGCCCAGAGAGTGACC
IFN-a4-R	GGCCCTCTTGTTCCCGAGGT
IFN-a5-F	CCTCAGGAACAAGAGAGCCTTA
IFN-a5-R	TCCTGTGGGAATCCAAAGTC
IFN-β-F	AGATGTCCTCAACTGCTCTC
IFN-β-R	AGATTCACTACCAGTCCCAG
IFIT2-F	TGGGGAAACTATGCTTGGGT
IFIT2-R	CCTCACAGTCAAGAGCAGGA
IIGP1-F	AATACCTGCCTCACGCTCAT
IIGP1-R	GCTACTCTGTGGGTTCTGGC
STAT1-F	CAGGTGTTGTCAGATCGAACCTTCC
STAT1-R	TTCAGCTCTTGCAATTTCACCAACA
NF-κB (p65)-F	CATTTCCGCCTCTGGCGAATG
NF-κB (p65)-R	CGTTGCTTCGGCTGTTCGATG
TNF-α-F	TCACTGGAGCCTCGAATGTC
TNF-α-R	GTGAGGAAGGCTGTGCATTG
ZO-1-F	GTCCCTCCTCTGATACCTTCCTC
ZO-1-R	CTGGCAGTGTCATTCACATCTTTCT
Occludin-F	TACAGACCCAAGAGCAGCA
Occludin-R	AGCCGTACATAGATCCAGAA
β-actin-F	CACTGCCGCATCCTCTTCCTCCC
β-actin-R	CAATAGTGATGACCTGGCCGT
